# Neuro‐Metabolic Association in Alzheimer’s Disease and Obesity: Clinical Evaluation of NA‐831 and NA‐931

**DOI:** 10.1002/alz70861_108265

**Published:** 2025-12-23

**Authors:** Lloyd Tran

**Affiliations:** ^1^ Biomed Industries, Inc., San Jose, CA USA

## Abstract

**Background:**

This study explores the potential link between Alzheimer’s disease (AD) and obesity by evaluating two oral drugs: NA‐831 is a novel drug candidate with neuroprotective, neurogenic, and memory‐enhancing properties for the treatment of Alzheimer’s Disease (AD). NA‐931, an analog of NA‐831, targets IGF‐1, GLP‐1, and GIP pathways to treat obesity.

**Method:**

NA‐831 for Alzheimer’s Disease: In a Phase 2 randomized clinical trial (ClinicalTrials.gov ID: NCT03538522), 112 participants with mild or moderate AD received either NA‐831 or placebo. Patients with mild cognitive impairment (MCI) received 10 mg/day, while those with mild to moderate AD received 30 mg/day. Inclusion criteria followed NIA‐AA standards, with MMSE scores ≥22 for MCI and 17–21 for mild to moderate AD.

NA‐931 for Obesity: A randomized, double‐blind, placebo‐controlled, dose‐escalation, first‐in‐human study (ClinicalTrials.gov ID: NCT06615700) was conducted to evaluate the safety, tolerability, pharmacokinetics, and pharmacodynamics of NA‐931. The study involved overweight/obese participants receiving single and multiple ascending doses, as well as multiple‐dose administration in patients with Type 2 Diabetes Mellitus.

**Result:**

NA‐831 for AD: After 24 weeks, NA‐831 treatment resulted in a significant cognitive improvement in AD patients, with a mean 4.1‐point improvement on the ADAS‐Cog‐13 scale compared to placebo (p = 0.001). Additionally, 78% of patients demonstrated clinical improvement on the CIBIC‐Plus scale (p = 0.01). NA‐831 was well tolerated at 30 mg/day, with no serious adverse events reported.

NA‐931 for Obesity: NA‐931 induced dose‐dependent weight loss over 28 days. Participants receiving 150 mg/day lost up to 6.8% of body weight, representing a 5.1% greater reduction compared to placebo (p < 0.001). During the 8‐week open‐label extension, weight loss was sustained, with reductions reaching 12.7% (10.4% over placebo). NA‐931 was well tolerated across all study phases. Mild nausea and diarrhea were each reported in 8.3% of participants at the highest dose (overall incidence: 3.7%). In the 12‐week extension, nausea occurred in 16.6% and diarrhea in 8.3% of participants. No vomiting was observed.

**Conclusion:**

The clinical findings of NA‐831 and NA‐931 support a potential mechanistic link between Alzheimer’s disease and metabolic disorders such as obesity and diabetes. While these results are encouraging, further studies are warranted to elucidate whether this association is causal.

